# Amino Acid Reduction Can Help to Improve the Identification of Antimicrobial Peptides and Their Functional Activities

**DOI:** 10.3389/fgene.2021.669328

**Published:** 2021-04-20

**Authors:** Gai-Fang Dong, Lei Zheng, Sheng-Hui Huang, Jing Gao, Yong-Chun Zuo

**Affiliations:** ^1^Inner Mongolia Autonomous Region Key Laboratory of Big Data Research and Application of Agriculture and Animal Husbandry, College of Computer and Information Engineering, Inner Mongolia Agricultural University, Hohhot, China; ^2^The State Key Laboratory of Reproductive Regulation and Breeding of Grassland Livestock, College of Life Sciences, Inner Mongolia University, Hohhot, China

**Keywords:** antimicrobial peptide, identification, reduced amino acid alphabet, two-stage classifier, supporting vector machine

## Abstract

Antimicrobial peptides (AMPs) are considered as potential substitutes of antibiotics in the field of new anti-infective drug design. There have been several machine learning algorithms and web servers in identifying AMPs and their functional activities. However, there is still room for improvement in prediction algorithms and feature extraction methods. The reduced amino acid (RAA) alphabet effectively solved the problems of simplifying protein complexity and recognizing the structure conservative region. This article goes into details about evaluating the performances of more than 5,000 amino acid reduced descriptors generated from 74 types of amino acid reduced alphabet in the first stage and the second stage to construct an excellent two-stage classifier, Identification of Antimicrobial Peptides by Reduced Amino Acid Cluster (iAMP-RAAC), for identifying AMPs and their functional activities, respectively. The results show that the first stage AMP classifier is able to achieve the accuracy of 97.21 and 97.11% for the training data set and independent test dataset. In the second stage, our classifier still shows good performance. At least three of the four metrics, sensitivity (SN), specificity (SP), accuracy (ACC), and Matthews correlation coefficient (MCC), exceed the calculation results in the literature. Further, the ANOVA with incremental feature selection (IFS) is used for feature selection to further improve prediction performance. The prediction performance is further improved after the feature selection of each stage. At last, a user-friendly web server, iAMP-RAAC, is established at http://bioinfor.imu.edu. cn/iampraac.

## Introduction

Antimicrobial peptides (AMPs) are a kind of special polypeptide substance which exists in living organisms (Bahar and Ren, 2013; Khamis et al., 2015; Lv et al., 2021a). It has a wide range of biological functions, such as broad antibacterial spectrum, high antibacterial activity and difficult to produce drug resistance (O’Brien-Simpson et al., 2018; Shoombuatong et al., 2018; Qin et al., 2019). In particular, it has almost no toxic effect on normal cells of higher animals, and can specifically inhibit the growth of certain target tumor cells. In addition, AMPs have multiple advantages such as the diversity of protein molecular quaternary structure and physicochemical properties. Therefore, AMPs have become research focus in the fields of animal and human medicine (Hancock and Sahl, 2006; Popovic et al., 2012; O’Brien-Simpson et al., 2018; Lv et al., 2021a), nutrition, food science, and immunology. The utilization of biological AMPs is expected to become an ideal way to solve the problem of drug-resistant bacteria.

The identification of experimental method for biological peptides is time-consuming and expensive, while computational method can assist in the AMPs prediction and their antibacterial activities classification. In the past decade, some machine learning methods (Lata et al., 2007, 2010; Chen et al., 2016; Akbar et al., 2017; Manavalan et al., 2017, 2018; Kabir et al., 2018; Yang et al., 2021) have been developed to recognize AMPs, such as k nearest neighbor method, random forest (Manavalan et al., 2018; Chung et al., 2019), and support vector machine (SVM) (Hajisharifi et al., 2014; Li and Wang, 2016; Meher et al., 2017; Zhang et al., 2021). In recent years, the recognition of AMPs is not limited to the problem of whether they are AMPs. Scientist begins to focus on recognition of antimicrobial activities (Xiao et al., 2013; Lin and Xu, 2016; Wang et al., 2017; Chung et al., 2019). Xiao used an improved fuzzy k-nearest neighbor method to determine which functional type this peptide belongs to (Xiao et al., 2013). Xu et al. adopted the oversampling method to improve the classification accuracy based on same dataset (Lin and Xu, 2016). In the past 3 years, models based on deep learning are gradually developed (Veltri et al., 2018; Fang et al., 2019; Zeng et al., 2019) for AMPs prediction, and better results have been achieved.

A good prediction method must be combined with an effective feature extraction scheme to achieve better prediction results. At present, there are many popular feature extraction schemes, including amino acid composition (AAC) (Li and Wang, 2016; Meher et al., 2017; Chung et al., 2019; Lv et al., 2019a,b), pseudo amino acid composition (PseAAC) (Shen and Chou, 2008; Khosraviana et al., 2013; Hajisharifi et al., 2014; Zare et al., 2015), physicochemical properties (Melo et al., 2011; Shua et al., 2013; Agrawal et al., 2018; Bhadra et al., 2018; Chung et al., 2019; Schaduangrat et al., 2019; Lv et al., 2020a; Zhang et al., 2020), binary position map (Chung et al., 2019), position specific scoring matrix (PSSM) (An et al., 2019; Kong and Zhang, 2019; Wang et al., 2019; Zhou et al., 2019; Zhu et al., 2019), gene ontology method (GO) (Camon et al., 2003; Wan et al., 2013; Zhou et al., 2017; Cheng et al., 2018), reduced amino acid (RAA) (Zuo et al., 2015, 2019; Zheng et al., 2019). For example, Lee introduced the concept of n-gram (Chung et al., 2019), calculated the features in n-gram using binary location map, and used the feature selection method for multi feature fusion, which has achieved good results in the classification practice of seven kinds of AMPs. Nalini Schaduangrat used the feature extraction method of amphiphilic pseudo amino acids composition (Schaduangrat et al., 2019) Am-PseAAC to predict anti-cancer peptides, and achieved a total accuracy of 95.61%.

The simplified amino acid alphabet is to reduce the alphabet of 20 natural amino acids to 2–19 groups by using different amino acid reduction methods (Zuo et al., 2017; Zheng et al., 2020). It not only includes physicochemical difference, such as hydrophilicity, hydrophobicity, polarity, charge, etc., but also contains a series of mathematical methods to simplify the natural amino acid alphabet, such as the number of residue types (Pape et al., 2010), the distances between amino acids (Wang and Wang, 1999), the perspective of evolution (Nanni and Lumini, 2008). Markov process, corresponding instantaneous replacement rate matrix (Kosiol et al., 2004), the conditional probability deviation from the random background (Liu et al., 2002),etc. Using a simplified alphabet can reduce the complexity of protein sequences while retaining the key information encoded in the sequences.

Therefore, in this paper, in order to improve the prediction performance of AMPs and their functional activities, there are 5,032 RAA descriptors are generated and computed based on RAACBook (Zheng et al., 2019). Furthermore, the amino acid reduction classifier for identifying AMPs and their activities is constructed. Finally, a freely accessed two-stage web server, named iAMP-RAAC, is build. In the first stage, whether an input sequence is an AMP is calculated, and its functional activity type is further predicted in the second stage. The results show that our classifier achieves good prediction performance both in the first stage and the second stage.

## Materials and Methods

**FIGURE 1:**
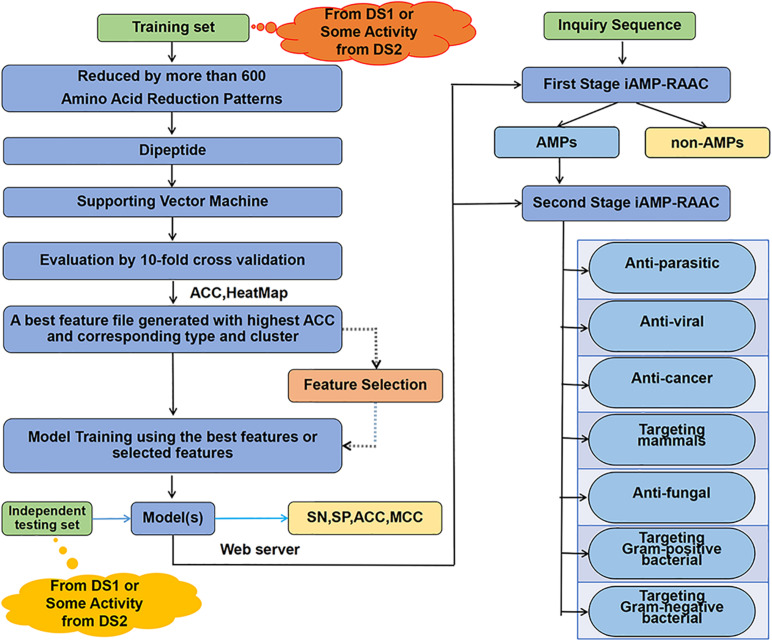
The overall framework of our classifier. Training data set from DS1 or seven training data sets from DS2 are computed separately through amino acid reduction, dipeptide feature extraction, supporting vector machine model training and 10-fold cross-validation model evaluation. Then, the best feature file with the highest accuracy and the corresponding reduction type and cluster are determined. Next, the best features after feature selection or features from the best feature file are used for model training. Finally, on the one hand, the independent test set is used for testing performances of model; on the other hand, the web server is constructed with the trained model to provide two-stage prediction service.

In order to clarify clearly the research ideas used in this paper, we draw the flow chart of our two-stage classifier as Figure 1. The details of the flowchart are described step by step in this chapter sections.

### Benchmark Dataset

The number of peptides with experimentally confirmed antimicrobial activities is very small. Thus, selecting proper negative samples for training is a challenge of building the benchmark dataset. To solve this challenge, a distance based method was proposed to select negative samples for constructing a high quality benchmark dataset by Chen (Chen et al., 2018). By using this method, the representative negative samples could be obtained by calculating the Euclidean distance.

In this work, for the comparison convenience, we use dataset the same as that in literature (Chung et al., 2019). It has two sets of data. DS1 is used in the first stage classifier, which is composed of training set and independent test set. The specific construction method is as follows: firstly, 6,766 positive sequences were downloaded from various data sources (Tyagi et al., 2013, 2015; Mehta et al., 2014; Qureshi et al., 2014; Lee et al., 2015; Fan et al., 2016; Wang et al., 2016; Manavalan et al., 2017; Agrawal et al., 2018); secondly, the sequences of lengths ranging from 5 to 255 were collected from AmPEP and UniProt, and the unnatural amino acids B, J, O, U, X, and Z were filtered; thirdly, the CD-HIT (Li and Godzik, 2006) and CD-HIT-2D (Li and Godzik, 2006) were used successively to delete the homologous sequences in the positive and negative data sets with a threshold of 50% identity; finally, 70% of the sequences in the positive and negative data set were used as the training set, including 1,686 positive and 16,428 negative samples respectively, and the other 30% of the sequences were taken as independent test sets, including 723 positive and 7,041 negative samples respectively.

DS2 is the data set of the second stage classifier. It consists of 7 training sets and 7 independent test sets corresponding to 7 different AMPs activities respectively, as shown in Table 1. Firstly, positive sample sequences were downloaded from multiple AMP databases (Chung et al., 2019). If a sequence has some activity, then put it in the positive set of that activity; at the same time put it in negative sets of other activities. The data sets of 7 AMPs activities were constructed in the same way. Then, 70% of the 7 data sets were randomly selected as training set and 30% as independent test set. Finally, CD-HIT-2D (Li and Godzik, 2006) was used to remove homologous and redundant sequences with a threshold of 50% identity.

**TABLE 1:** The Number of AMPs of seven AMP functional activities on training set and testing set for DS1 and DS2.

Activities	Positive samples (training/testing)	Negative samples (training/testing)
Anti-parasitic	140/60	700/1,914
Anti-viral	1,400/601	2,451/1,374
Anti-cancer	219/94	1,095/1,881
Targeting mammals	215/93	1,075/1,882
Anti-fungal	1,912/820	1,261/1,155
TGPB	1,930/828	1,624/1,147
TGNB	1,931/828	1,635/1,147

*“TGPB” means Targeting Gram-positive bacteria; “TGNB” means Targeting Gram-negative bacteria.*

### Feature Extraction

The RAACBook (Zheng et al., 2019) provides 74 kinds of amino acid reduction types. Each type can produce up to 18 different reduction clusters between 2 and 19. For the training datasets in DS1 and DS2, 629 amino acid reduced descriptors were generated after removing the repetitive ones in the first stage, and 4,403 (629 × 7) amino acid reduced descriptors were generated after removing the repetitive ones in the second stage. So, there are a total of 5,032 amino acid reduced descriptors in our classifier. The input sequences are computed by the amino acid reduction descriptors and dipeptide composition successively. For example, for the AMP sequence:

> ap00006 GNNRPVYIPQPRPPHPRI

Supposing the reduction type 1, i.e., BLOSUM50 matrix, it could generate 10 different amino acid reduction descriptors. The 10 cluster sizes, the clusters and sequences after reduction are shown in Table 2. If cluster size equals to 2, then the other amino acid will be replaced by the first amino acid “L” or “E” in “LVIMCAGSTPFYW” or “EDNQKRH”. The methods of other cluster sizes for reducing process are similar.

**TABLE 2:** Reduction descriptors when reduced type is 1 and cluster size are 2–19.

Cluster Size	Reduced amino acid cluster	Sequence after reduction
2	LVIMCAGSTPFYW-EDNQKRH	LEEELLLLLELELLELEL
3	LASGVTIPMC-EKRDNQH-FYW	LEEELLFLLELELLELEL
4	LVIMC-AGSTP-FYW-EDNQKRH	AEEEALFLAEAEAAEAEL
5	LVIMC-AGSTP-FYW-EDNQ-KRH	AEEKALFLAEAKAAKAKL
6	LVIM-AGST-PHC-FYW-EDNQ-KR	AEEKPLFLPEPKPPPPKL
8	LVIMC-AG-ST-P-FYW-EDNQ-KR-H	AEEKPLFLPEPKPPHPKL
10	LVIM-C-A-G-ST-P-FYW-EDNQ-KR-H	GEEKPLFLPEPKPPHPKL
12	LVIM-C-A-G-ST-P-FY-W-EQ-DN-KR-H	GDDKPLFLPEPKPPHPKL
15	LVIM-C-A-G-S-T-P-FY-W-E-D-N-Q- KR-H	GNNKPLFLPQPKPPHPKL
18	LM-VI-C-A-G-S-T-P-F-Y-W-E-D-N-Q-K-R-H	GNNRPVYVPQPRPPHPRV
20	L-V-I-M-C-A-G-S-T-P-F-Y-W-E-D-N-Q-K-R-H	GNNRPVYIPQPRPPHPRI (original sequence)

Dipeptide composition is widely used in protein feature extraction, and its calculation method is as Formula (1). *N* is the length of an input sequence, *p*_*i*_ or *p*_*j*_ is a kind of amino acid from 20 natural amino acids, and *Num*(*p_*i*_ p_*j*_*) represents the number of string *p_*i*_ p_*j*_*.

(1) $$\text{Com}\,\left(p_{i}\,p_{j}\right)=\frac{N\,u\,m\,\left(p_{i}\,p_{j}\right)}{N-1}$$

### Model Construction

This paper constructed a two-stage classifier, iAMP-RAAC. In the first stage, a binary classification model was constructed, and in the second stage, 7 binary classification models corresponding 7 antimicrobial activities were constructed. So we have a total of eight models. SVM is an outstanding model in machine learning algorithms, so in our study, we adopt this model for training and evaluation of the 8 models. In order to achieve competitive performance, we use gauss kernel function and grid search strategy for getting the best super parameters. The searching ranges of super parameter gamma, C are shown as formula (2).

(2) $$\{\begin{array}{c} 2^{-n}\leq \text{gamma}\leq 2^{n}\\ 2^{-n}\leq C\leq 2^{n} \end{array}$$

### Performance Evaluation

We use sensitivity (SN), specificity (SP), accuracy (ACC), Matthews correlation coefficient (MCC) to measure the quality of the classifier for DS1 and DS2 (Amanat et al., 2020; Chen et al., 2020; Ikram et al., 2020; Ilyas et al., 2020; Kong et al., 2020; Liang and Zhang, 2020; Lv et al., 2020b, 2021b). The calculation formula is as formula (3).

(3) $$\left\{ \begin{array}{c} S\,N=\frac{T\,P}{T\,P+T\,N}\\ S\,P=\frac{T\,N}{T\,N+F\,P}\\ A\,C\,C=\frac{T\,P+T\,N}{T\,P+T\,N+F\,P+F\,N}\\ M\,C\,C=\frac{\left(T\,P^{*}\,T\,N\right)-\left(F\,P^{*}\,F\,N\right)}{\sqrt{\left(T\,P+F\,P\right)\,\left(T\,P+F\,N\right)\,\left(T\,N+F\,P\right)\,\left(T\,N+F\,N\right)}} \end{array}\right.$$

Where TP, true positives, represents the number of positive samples correctly predicted.TN, true negatives, indicates the number of correctly predicted negative samples. FP, false positives, represents the number of positive samples predicted incorrectly. FN, false negatives, indicates the number of negative samples predicted incorrectly (Patil and Chouhan, 2019; Long et al., 2020; Lv et al., 2020c, 2021c; Smolarczyk et al., 2020; Tahir and Idris, 2020; Tripathi et al., 2020; Wang et al., 2020; Zhu et al., 2020).

### Feature Selection

Protein prediction is very similar to text classification. The commonly used feature selection methods in text classification, such as ANOVA and Chi-Square Test, have the defect of favoring low-frequency words. But dipeptide feature extraction method makes up for this defect. So, in this paper, ANOVA and incremental feature selection (IFS) were employed to extract useful features to improve prediction performance (Feng et al., 2019). Firstly, ANOVA was used to compute the variance values of all features; secondly, sort the features according to the values of ANOVA; finally, the best *n* features are determined by adding features step by step according to a preset step size.

### Model Validation

Among the three validation methods of jackknife validation, k-fold cross validation and independent test set validation, jackknife is recognized as the most objective and rigorous cross validation method, because its calculation results are always unique. However, in order to compare with the results of literature, this paper uses 10-fold cross validation to train model and uses independent test set to evaluate model.

### Webserver Development

An interface friendly webserver was developed with classifier iAMP-RAAC embedded. People can freely access the website and compute an/inquiring peptide(s). The address of the webserver is http://bioinfor.imu.edu.cn/iampraac.

## Results and Discussion

### Performance Evaluation for AMPs and Non-AMPs

We firstly evaluate the four predictors that trained based on the training set in DS1 by 10-fold cross-validation and list the results in Table 3. It can be seen that iAMP-RAAC obtains the maximum SP, ACC, and MCC of 98.94, 97.21, and 82.84% with 361 features respectively, while AMPfun got the ACC of 95.09% with 9,367 features. There are two reasons for the improvement of performance. On one hand, the application of Gaussian kernel function of SVM and the search strategy of hyper parameter makes model find best parameters (Gamma = 2, C = 2); on the other hand, the amino acid sequence with appropriate reduction contains more refined and useful features. Thus, the ACC of iAMP-RAAC exceeds 2.12% of that by AMPfun, conversely, the number of features is only 3.85% of that by AMPfun.

**TABLE 3:** Performance comparisons of iAMP-RAAC and the other three methods on training set in DS1 based on 10-fold cross-validation.

Method	SN (%)	SP (%)	ACC of BFS/ACC of AFS	MCC (%)	Number of features for BFS/number of features for AFS
iAMP-RAAC	84.30	98.94	97.21%/ 97.23%	82.84	361/336
AMPfun (Chung et al., 2019)	94.88	95.11	95.09%/−	77.06	9,367/2,452
SVM	94.33	94.29	94.3%/−	74.47	−/−
DT	83.40	98.26	96.87%/−	81.47	−/−

*“−” means that there is no value in the corresponding item; “BFS” means Before Feature Selection and “AFS” means After Feature Selection. N(BFS) means number of features BFS; N(AFS) means number of features AFS.*

**FIGURE 2:**
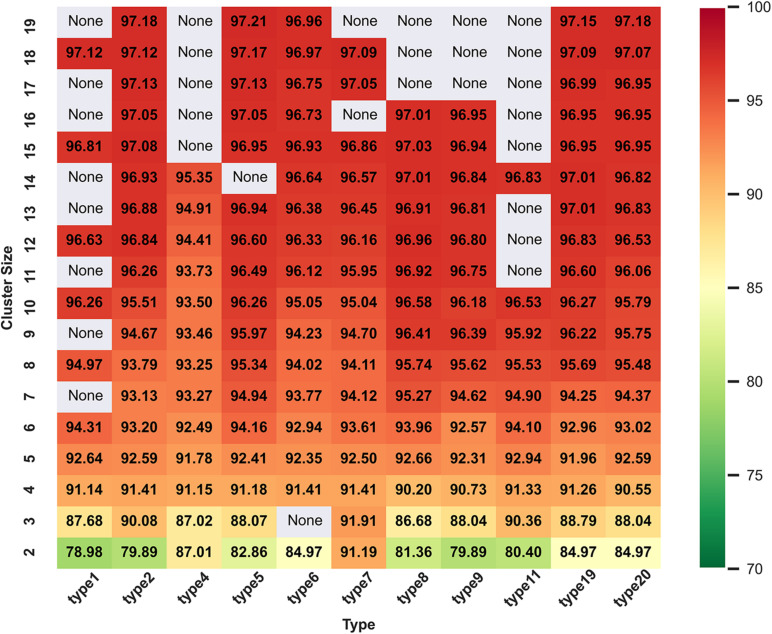
Heat map of ACC values with reduced types from 1 to 20 and cluster size of 2 to 19 on training dataset in DS1. In general, the color gradient from green to red indicates the increasing trend of the values of ACC, and the areas with “None” indicate that there are no such reduction descriptors at the intersections of the corresponding reduction types and cluster sizes.

Figure 2 and Supplementary Figure 1 show all ACC values from cluster size 2 to 19 in range of amino acid reduction type 1 to type 20. When reduced type is 5 and cluster size is 19, classifier gets the best accuracy of 97.21%. Here, a fact needs to be state that we have calculated all the 629 descriptors of 74 types separately and they are 1–20, 21–40, 41–60, and 61–74, respectively. Since the highest ACC appears in type 5 and cluster size 19, only the heat map and histogram of type 1 to 20 are shown. It can be seen that the expression of histogram and heat map are consistent and when the cluster size is more than 10, the classification performance will be significantly improved. This may be because if the size of the cluster is too small, it is hard to express all the information of the sequence.

**FIGURE 3:**
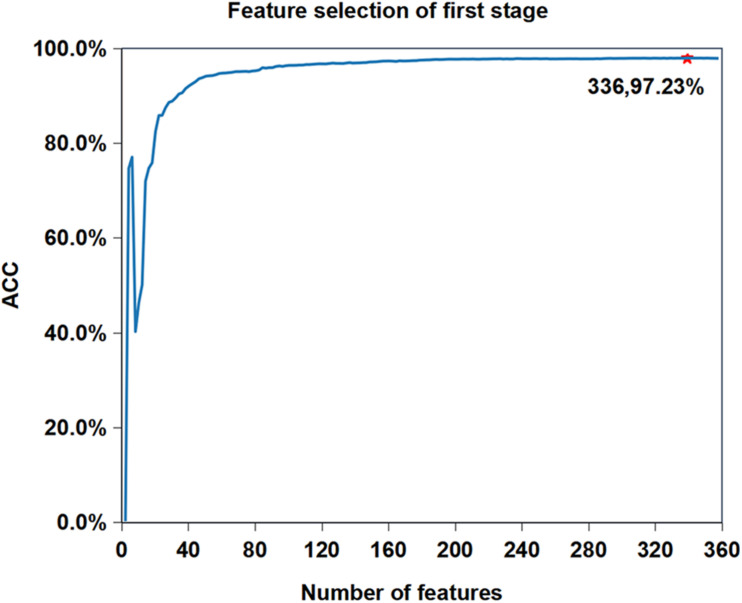
Feature selection process when reduction type is 5 and cluster size is 19 in the first stage on training set in DS1. The horizontal axis represents the number of features, and the vertical axis represents the value of ACC. The number of selected features and the value of corresponding ACC are marked on the curve.

We want to know whether the prediction performance will be further improved after feature selection based on the current best performance (Reduction type = 5, Cluster size = 19). Figure 3 shows the feature selection process when cluster size is 19 and reduced type is 5. We can see that the accuracy of iAMP-RAAC is improved from 97.21 to 97.23%, and the number of features is reduced from 361 to 336. Although AMPfun reduced the number of features from 9,367 to 2,452 after feature selection, compared with iAMP-RAAC, the latter is only 13.70% of the former. This result proves that combination of ANOVA and IFS is an effective method to filter useful features.

We compare the performance of iAMP-RAAC and AMPfun on independent test set. As seen in Table 4, AMPfun acquired AUC of 98.94% by 2,452 features, while iAMP-RAAC gets that of 98.47% by only 361 features. Although AMPfun didn’t calculate SN, SP, ACC and MCC, we find that the evaluation metric values on independent test set are lower than that on training set for most datasets in general. Because the SP, ACC and MCC of iAMP-RAAC on the independent test set are higher than those on the training set of AMPfun, therefore, we believe metric values of iAMP-RAAC performs better than that of AMPfun on the independent test set.

**TABLE 4:** Performance comparisons of iAMP-RAAC and the other method on independent test set in DS1.

Method	SN (%)	SP (%)	ACC (%)	MCC (%)	AUC (%)	Number of Features
iAMP-RAAC	88.44	97.91	97.11	82.24	98.47	361
AMPfun	–	–	–	–	98.94	2,452

*“–” means that there is no value in the corresponding item.*

### Performance Evaluation of AMPs With Various Functional Activities

In order to investigate the classification performance of seven different antimicrobial functional activity classifiers on the training set in DS2, we evaluate RF and iAMP-RAAC. As shown in Table 5, except anti-viral, each ACC and MCC of iAMP-RAAC exceed RF, especially ACC of anticancer peptides exceed 15% of that of RF, and MCC of targeting mammals exceed 36% of that of RF. Although the performances of SN for several activities are lower than that of RF, iAMP-RAAC performs better than RF as a whole. It may also imply that any model is not perfect and each has its own advantages and disadvantages.

**TABLE 5:** Performance comparisons of iAMP-RAAC and RF (Chung et al., 2019) on training set in DS2 in the seven different AMP functional activities based on 10-fold cross-validation.

Activity	Method	SN (%)	SP (%)	ACC (%)	MCC (%)
Anti-parasitic	iAMP-RAAC	50.00	96.43	88.69	54.65
	RF	75.26	83.66	82.02	49.55
Anti-viral	iAMP-RAAC	88.21	94.70	92.34	83.41
	RF	91.09	93.24	92.47	83.82
Anti-cancer	iAMP-RAAC	52.12	97.99	90.34	61.19
	RF	76.73	78.88	78.55	45.07
Targeting mammals	iAMP-RAAC	69.72	96.93	92.40	71.20
	RF	86.77	88.93	88.53	66.20
Anti-fungal	iAMP-RAAC	91.27	78.58	86.23	71.04
	RF	85.73	85.53	85.65	70.50
TGPB	iAMP-RAAC	89.90	88.61	89.31	78.51
	RF	88.52	88.48	88.51	76.87
TGNB	iAMP-RAAC	90.58	87.83	89.32	78.50
	RF	88.05	88.15	88.09	76.06

**FIGURE 4:**
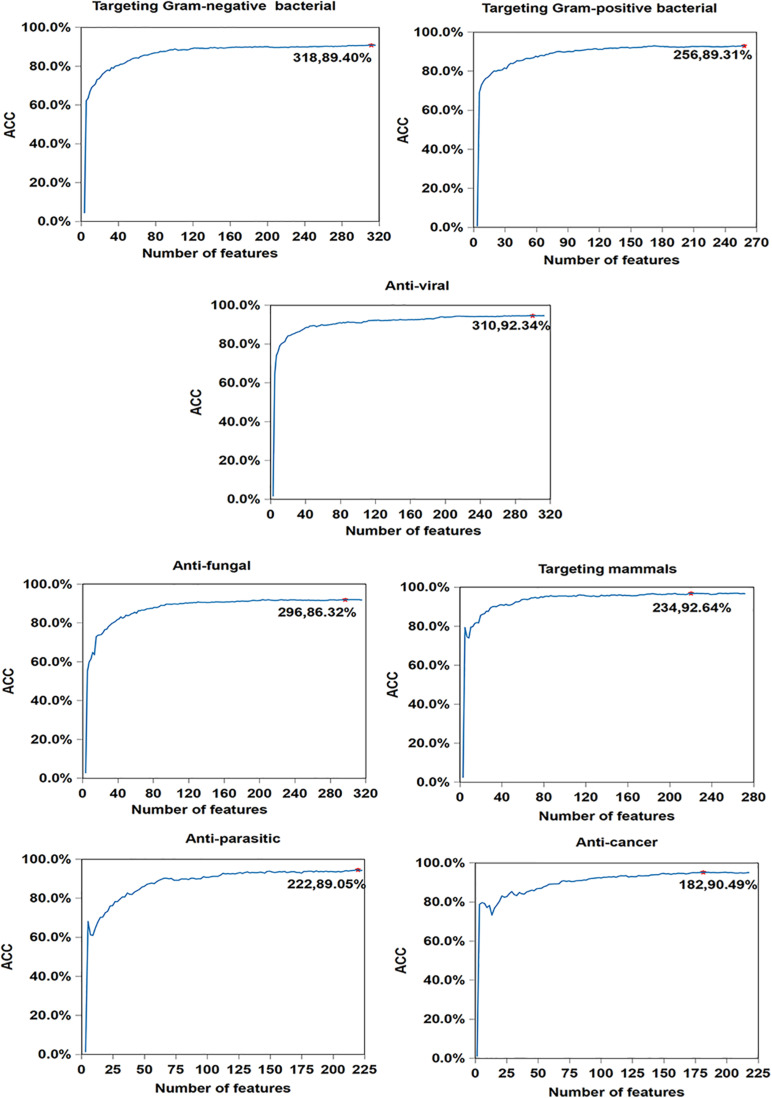
Result of feature selections for seven AMP functional activities. The horizontal axis represents the number of features, and the vertical axis represents the value of ACC. The number of selected features and the value of corresponding ACC are marked on the curve.

In order to illustrate the effectiveness of feature selection, we make corresponding feature selections after obtaining the optimal type and corresponding cluster size (as is shown in Supplementary Table 1) of 7 antimicrobial activities. As seen in Figure 4, compared with Table 5, the accuracy of anticancer peptides increases from 90.34 to 90.49%, and the number of features decreases from 225 to 182. It is similar with antifungal peptides, Gram-negative bacteria, targeting mammals, and anti-parasitic peptides. Overall, although the improvement is small, the feature selection process guarantees the minimum number of features and the maximum accuracy of each functional activity of AMPs.

To validate robustness of our model, iAMP-RAAC is further compared with other prediction tools on independent test set, such as AMPfun, iAMPpred, AVPpred, and MLACP. The performances of iAMP-RAAC and other methods with respect to various functional activities on the independent test set are displayed in Table 6. Overall, iAMP-RAAC achieves much higher SP, ACC and MCC values for all functional activities than other methods, for example, the values of SP for iAMP-RAAC almost all exceed 90.00% except that of Targeting Gram-negative bacterial, and are much higher than other methods. Our ACC values are 15.44 and 20.60% higher than those of AMPfun for anti-parasitic and anti-cancer peptides, while the values of SN are not so good. This is consistent with the comparison results on the training set in DS1.

**TABLE 6:** Performance comparisons of iAMP-RAAC and other methods on independent test set in DS2 in the seven different AMP functional activities.

Activity	Method	SN (%)	SP (%)	ACC (%)	MCC (%)
Anti-parasitic	iAMP-RAAC	14.10	97.91	91.29	18.88
	AMPfun	61.67	77.32	76.85	15.70
Anti-viral	iAMP-RAAC	76.64	95.05	88.51	74.58
	AMPfun	90.85	84.06	86.13	70.75
	iAMPpred (Xiao et al., 2013)	31.28	39.59	37.06	-26.82
	AVPpred (Thakur et al., 2012)	24.09	88.57	69.01	16.43
Anti-cancer	iAMP-RAAC	30.48	97.93	91.54	39.07
	AMPfun	77.66	70.60	70.94	22.08
	MLACP (Manavalan et al., 2017)	72.34	75.12	74.99	22.72
Targeting mammals	iAMP-RAAC	25.66	98.00	89.72	35.56
	AMPfun	78.49	80.45	80.35	29.98
Anti-fungal	iAMP-RAAC	63.61	91.21	74.73	54.57
	AMPfun	85.61	66.75	74.58	51.86
	iAMPpred (Xiao et al., 2013)	66.10	72.12	69.62	37.96
TGPB	iAMP-RAAC	67.03	90.09	77.16	57.45
	AMPfun	88.77	63.73	74.23	52.54
TGNB	iAMP-RAAC	68.28	89.37	77.92	58.21
	AMPfun	85.75	65.74	74.13	51.16

### Case Study

We obtained the data set of 1,028 anti-fungal peptides by searching anti-fungal peptides in UniProt database as an example to further illustrate the usability of our classifier. These 1,028 anti-fungal peptides took less than a minute to calculate at our webserver, and 892 of them were correctly identified. However, the AMPfun does not support uploading files composed of batch sequences. It can only paste sequences in FASTA format into the input box and the format is strict, so, it is difficult to calculate results successfully. For iAMPpred, it takes about 1 m to predict a sequence and can’t predict more than five sequences at a time, so it may be not practical.

## Conclusion

In this work, a two-stage classifier was constructed by pre-processing the input sequences with 5,032 amino acid reduction descriptors to complete the prediction of AMPs and their functional activities. The hybrid of amino acid reduction can significantly improve the prediction performance of the classifier. Whether on training set or on independent test set, whether AMPs or their functional activities, the prediction accuracy of the classifiers exceed almost all those in the existing literature. The feature selection process made it possible to obtain the best prediction accuracy values by using the least number of features. Further, by calculating all clusters of all reduction types, the best amino acid reduction types and cluster sizes for AMPs and their functional activities were obtained. According to the biological significance of some specific reduction type and their cluster found, biologists will be able to design new anti-infective drugs with fine granularity to AMPs and some specific activity. In the future, we will further analyse the importance features to find the correlation between characteristics and activities. In addition, the combination of amino acid reduction and graph neural network or other deep learning methods (Dao et al., 2020; Wang et al., 2021) is also considered to further improve the prediction performances.

## Data Availability Statement

The original contributions presented in the study are included in the article/Supplementary Material, further inquiries can be directed to the corresponding authors.

## Author Contributions

G-FD carried out the computation and wrote the manuscript. LZ designed and developed the webserver. S-HH programmed the algorithm. JG conceived the selection of feature parameters. Y-CZ planned overall and performed the results analysis. All authors reviewed the manuscript.

## Conflict of Interest

The authors declare that the research was conducted in the absence of any commercial or financial relationships that could be construed as a potential conflict of interest.

## Supplementary Material

Supplementary Figure 1 Evaluating bar chart of accuracy (ACC) values for reduced types ranging from 1 to 20 and cluster size of 2 to 19 on training dataset in DS1. The columns of corresponding reduced type and cluster size with highest ACC are marked with the highest ACC values. For example, the highest ACC value 97.21% is marked on the columns of the fifth reduced type and the 19th cluster size.

Supplementary Table 1 The hyper parameters of SVM, the best type, and the corresponding cluster size of seven different AMP functional activities.
