# Supplementary figures and images for: Amino Acid Reduction Can Help to Improve the Identification of Antimicrobial Peptides and Their Functional Activities
Source: Front Genet. 2021 Apr 20;12:669328. doi: 10.3389/fgene.2021.669328 (PMC8093877; doi:10.3389/fgene.2021.669328)

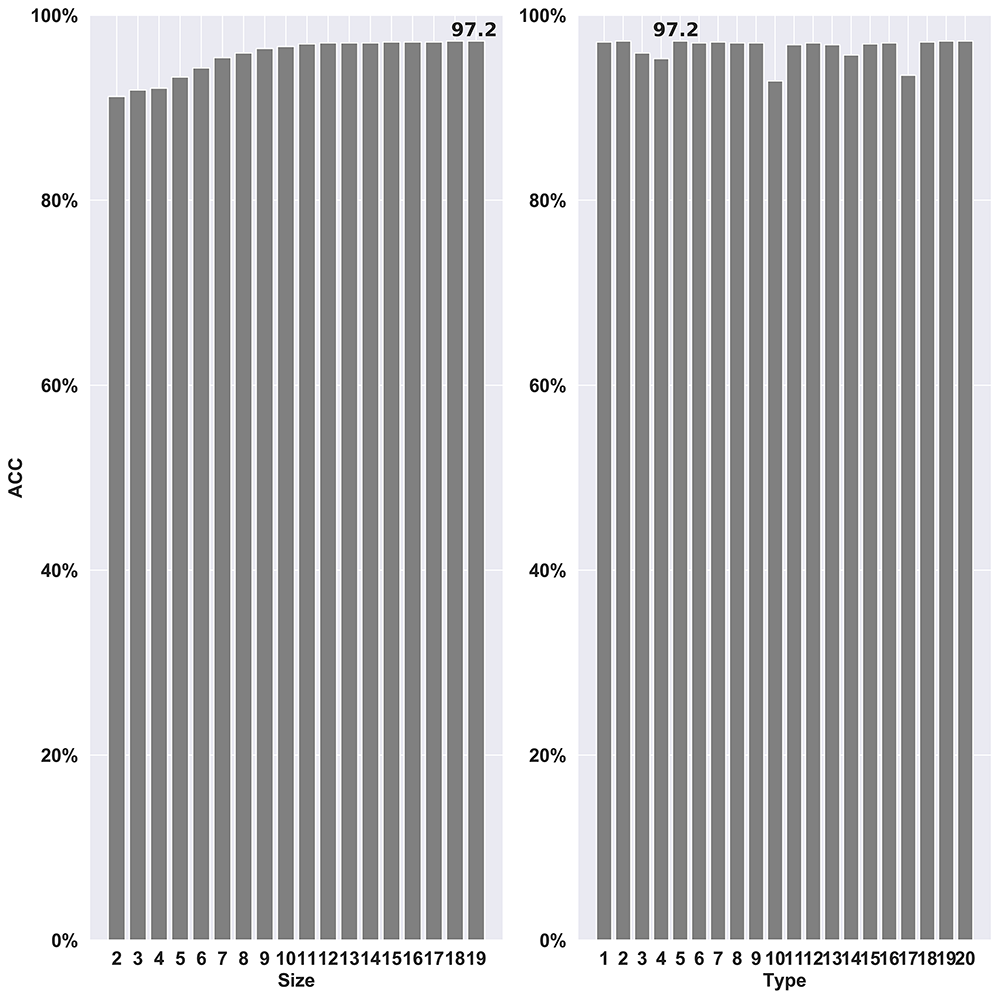

Supplement: Supplementary Figure 1 — Evaluating bar chart of accuracy (ACC) values for reduced types ranging from 1 to 20 and cluster size of 2 to 19 on training dataset in DS1. The columns of corresponding reduced type and cluster size with highest ACC are marked with the highest ACC values. For example, the highest ACC value 97.21% is marked on the columns of the fifth reduced type and the 19th cluster size. [file Image_1.TIF]
